# The role of the vomeronasal system in food preferences of the gray short-tailed opossum, *Monodelphis domestica*

**DOI:** 10.1186/1743-7075-2-6

**Published:** 2005-02-22

**Authors:** Mimi Halpern, Yasmine Daniels, Ido Zuri

**Affiliations:** 1 Department of Anatomy and Cell Biology, SUNY Downstate Medical Center, 450 Clarkson Avenue, Brooklyn, NY 11203 USA

## Abstract

Although feeding deficits have been reported in snakes and lizards following vomeronasal system disruption, no deficit has been previously reported in a mammal. We tested gray short-tailed opossums with items from four different food categories prior to occluding access to the vomeronasal organ. Preoperatively, opossums preferred meat to fruit or vegetables. Following occlusion of the nasopalatine canal, but not after control treatment, opossums failed to demonstrate food preferences.

## 

The vomeronasal system (VNS) is a nasal chemosensory system usually considered primarily a pheromone-detecting system [[1-4] for recent reviews]. The system, present in most terrestrial vertebrates, is comprised of the peripherally situated vomeronasal organ (VNO), whose sensory neurons project their axons to the accessory olfactory bulb (Figure 1), and the further projections from the accessory bulb into the limbic forebrain [1-4].

**Figure 1:**
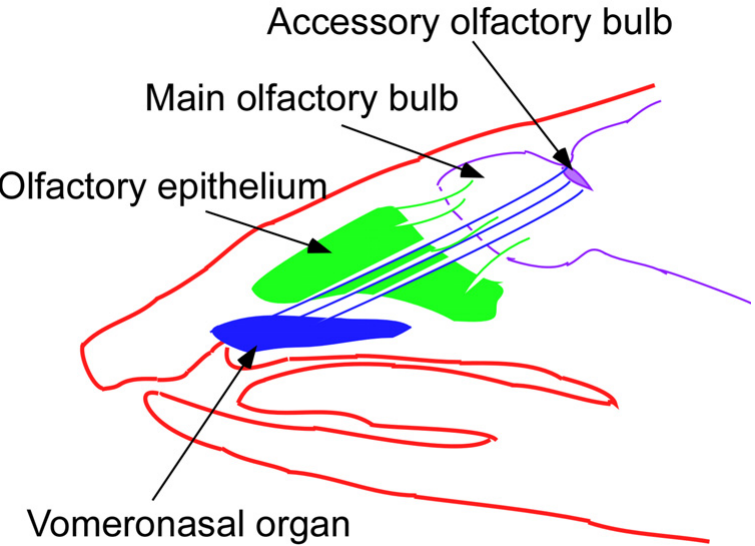
Head of an opossum as viewed from the side illustrating the position of the vomeronasal organ, olfactory epithelium and their respective connections to the accessory olfactory bulb and main olfactory bulb.

In snakes and lizards, in addition to its role in pheromone detection, the VNS is important for feeding behavior [5-7]. To date, no study has reported a feeding deficit in a mammal deprived of a functional VNS. It is possible that the failure to report feeding deficits in rats, mice and hamsters deprived of a functional VNS is a result of the fact that these animals have been fed laboratory diets over many generations. It is also conceivable that with the emphasis on the pheromonal function of the VNS, the issue of the role of the VNS in feeding behavior was overlooked.

Gray short-tailed opossums, *Monodelphis domestica*, are members of the Didelphidae or American opossums. The didelphids are the most ancient marsupial family and thought to be the origin of all other New World and Australian marsupials [8,9]. *M. domestica *was recently introduced into laboratories [10] and has, therefore, not been fed a laboratory diet over many generations. As these opossums respond to a variety of foods [11-13], they are good models to investigate the role of the VNS in food preferences in mammals.

Six male and seven female gray short-tailed opossums, 12–16 months old, were used as test subjects in this study. The opossums were progeny of animals originally purchased from the Southwest Foundation for Biomedical Research (San Antonio, Texas, USA) and were offspring of different parents. The opossums were housed individually in plastic cages (42 × 21 × 20 cm) with wood shavings as bedding, and provided with cylindrical plastic containers for nesting. The animals had dry fox food (Milk Specialties Co., New Holstein, WI, USA) and water available *ad libitum*.

To determine which foods to use in the formal experiment, a preliminary test was conducted in which 24 different foods, representing meats, fruits and vegetables were presented to the opossums. Each opossum was presented with one food per day, selected at random, and its consumption of the food noted. The animals were allowed three minutes to investigate and eat the food, which was placed on an 8 × 20 cm cardboard tray at the front of the home cage. Each animal received two trials with each food. Of the 24 foods tested 16 were selected for the formal experiment based on the animals' observed preferences.

During testing the opossums were simultaneously presented with four foods one from each of the following food groups: fruits (apples, oranges, peaches, cantaloupes), meats (mealworms, chicken, pork, crickets), processed vegetables (Raisin Bran, Cheerios, whole wheat bread, bagel) and unprocessed vegetables (corn, peppers, carrots, broccoli). The animals were tested daily with one of 16 food combinations (4 foods × 4 categories). Four sterile Petri dishes, each 3.5 × 1.0 cm, were placed equidistant from each other and glued to an 8 × 20 cm cardboard tray. One piece (0.21 cm^3^) of food from each food category, was placed in a dish.

Before each test session, feeding troughs containing fox food were removed from each opossum's cage. At the beginning of a trial the food tray was placed in the front of the animal's home cage and the behavior of the animal videotaped for three minutes using a digital video-camera (Sony DCR-VX2000, 30 frames/sec) placed at a distance of 3 m from the test cage. Trays, dishes and unconsumed food were discarded after each trial. After testing, food troughs containing fox food were replaced in the animals' home cages. Fresh disposable gloves were used when handling dishes, trays and foods to prevent transfer of food odors.

Videotapes of food trials were analyzed using a videocassette recorder (JVC SR-VS30U), BTV Pro 5.4.1 and "Videoanalyzer" software (designed by John L. Kubie, Downstate Medical Center).

For control surgery and occlusion of the nasopalatine canal, opossums were lightly anesthetized with Ketaset (0.25 cc/100 g, i.m.) and atropine sulfate (.05 cc/100 g, i.m.) Access to the vomeronasal organ (VNO) was blocked with gel foam and Crazy Glue™ (Elmer's Products, Inc., Columbus, OH) applied to the roof of the mouth, covering the opening to the nasopalatine canal. Cresyl violet crystals were added to the Crazy Glue to facilitate visualization of the block. Opossums were visually checked daily to insure that the VNO block remained in place. Control surgery consisted of placing Crazy Glue on the roof of the mouth to either side of the nasopalatine canal. Opossums were allowed two days to recover before postoperative testing.

The VNO block remained in place for only one test day in all thirteen opossums. Therefore, pre-post operative and control comparisons were only made for trials identical in composition (foods tested) and order (position on the tray) to the first postoperative test day. Control postoperative trials were run as described above. All 13 animals were tested under both control and experimental (nasopalatine canal blocked) conditions, with the order of surgical treatment randomized.

The first food selected and consumed on each trial was identified as the animal's food choice. Statistical analyses utilized a chi-square test of the frequencies of choices of each food type for pre-operative, control and experimental trials. Figure 2 depicts the results in terms of the percentage of choices of each food type under the three conditions.

**Figure 2:**
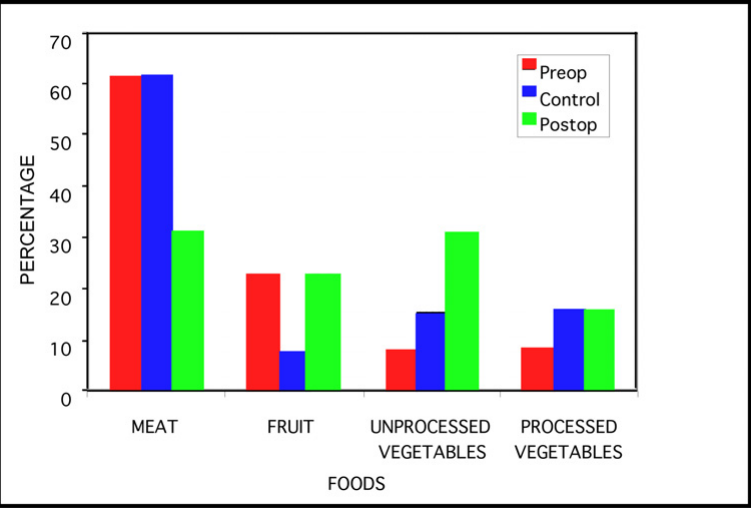
Percentage of food choice responses (first food type selected and consumed) by opossums prior to surgery (Preop), after control occlusion (Control) and after occlusion of the nasopalatine canal (Postop).

Preoperatively, opossums preferred meats to fruits and fruits to processed and unprocessed vegetables (Figure 2). The most preferred food within the meat category was crickets, within the fruit category was cantaloupe, within the unprocessed vegetable category was corn and within the processed vegetable category was whole wheat bread.

Whereas the preoperative comparison trials on the thirteen opossums revealed significant preferences among the food categories (χ^2 ^= 10.08, df = 3, p < .02), postoperatively, no significant difference in food category preference was observed (χ^2 ^= 0.85, df = 3, p > .05). During control trials the opossums continued to demonstrate significant food preferences (χ^2 ^= 9.46, df = 3, p < .05,). Meat was again, the most preferred food category (Figure 2).

One deficiency of this study is that, because the opossums removed the glue blocks after the first day, we were unable to verify that the nasopalatine occlusion had, indeed, prevented access to the vomeronasal organ. However, we had previously [14] demonstrated, using the identical technique, that this method prevented access of substances to the VNO. Furthermore, the failure of opossums with nasopalatine canal occlusion to demonstrate the food preferences observed during preoperative and under control conditions, strongly suggests that the block was effective.

This study suggests that without a functional VNS, the food preferences of gray short-tailed opossums are significantly impaired. Previous studies on the VNS of mammals have not addressed the issue of changes in feeding behavior. It remains to be seen whether other mammals, newly introduced into the laboratory, or bred over many generations in the laboratory, also demonstrate a food preference deficit when deprived of a functional VNS. Studies comparing wild populations with laboratory-reared populations might contribute information resolving this issue.

It is not surprising that food preferences might be influenced by vomeronasal stimulation since the vomeronasal organ of many vertebrates, including opossums, is directly accessible from the oral cavity [2] via the nasopalatine duct. Thus, food in the mouth could be sensed by the vomeronasal system. Whether this mechanism is, in fact, utilized in food selection is not known and would have to be the subject of future investigation. It is likely, however, that the expression of food preference is a result of the interaction of multiple sensory systems including taste, olfaction, vision, trigeminal and vomeronasal.

## Authors' contributions

MH conceived of and designed the experiment and wrote the manuscript

YD conducted the experiment, extracted the data from the videotapes and analyzed the data statistically

IZ supervised YD in the conduct of the experiment and instructed YD in the experimental procedures and data analysis
